# The Impact of Human–Robot Synchronization on Anthropomorphization

**DOI:** 10.3389/fpsyg.2018.02607

**Published:** 2019-01-08

**Authors:** Saskia Heijnen, Roy de Kleijn, Bernhard Hommel

**Affiliations:** ^1^Cognitive Psychology Unit, Leiden University, Leiden, Netherlands; ^2^Leiden Institute for Brain and Cognition, Leiden, Netherlands

**Keywords:** anthropomorphization, robot, synchrony, Simon task, Dictator Game, imitation, agency, self-other overlap

## Abstract

To elucidate the working mechanism behind anthropomorphism, this study investigated whether human participants would anthropomorphize a robot more if they move synchronously versus non-synchronously with it, and whether this is affected by which of the two initiates the movements. We tested two competing hypotheses. The feature-overlap hypothesis predicts that moving in synchrony would increase perceived self-other feature overlap, which in turn might spread activation to codes of features related to humans—which should increase anthropomorphization. In contrast, the autonomy hypothesis predicts that unpredictability increases anthropomorphization, and thus that whenever the robot initiates movements, or when the human initiates movements to which the robot moves non-synchronously, there is an increased perception of the robot as a more human-like, intentionally acting creature, which in turn should increase anthropomorphization. We performed a study with synchrony as within-subjects factor, and initiator (robot or human) as between-subjects factor. To study the impact of synchrony on self-other overlap and perception of human likeness, participants completed two tasks that served as implicit measures of state anthropomorphization, and two questionnaires that served as explicit measures of state anthropomorphization toward the robot. The two implicit measures were the joint Simon task and one-shot Dictator Game. Additionally, participants filled in a trait anthropomorphization questionnaire, to enable correction for baseline tendencies to anthropomorphize. The synchrony manipulation did not affect the joint Simon effect, although there was an effect on average reaction time (RT), where in the group in which the robot initiated the movement, RTs were slower when the human and robot moved non-synchronously. The Dictator Game offer and the state anthropomorphization questionnaires were not affected by the synchrony manipulation. There was, however, a positive correlation between current anthropomorphization of the robot and amount of money offered to it. Given that most measures were not systematically affected by our manipulation, it appears that either our design was suboptimal, or that synchronization does not affect the anthropomorphization of a robot.

## Introduction

Anthropomorphization is commonly defined as the attribution of human mental states and characteristics to non-human animals and objects. There are two components to anthropomorphization: attributing human physical features to non-human animals and objects (e.g., seeing a face in the clouds), and attributing a human mind to non-human animals and objects (Waytz et al., 2010). This attribution encompasses not only emotions (e.g., my cat is grumpy), but also higher-order mental states such as intentions, desires, self-reflection, consciousness, and agency (e.g., my cat is proud of the jump he just made). Anthropomorphization has a strong impact on how humans perceive, appreciate, and interact with artificial systems and robots in particular. Ample evidence suggests that a greater tendency to anthropomorphize—be it due to individual traits or situational factors—increases the acceptability of robots and the degree to which people enjoy interacting with and trust robots (for reviews, see Hancock et al., 2011; Fink, 2012; Zlotowski, 2015).

The present study investigated the effect of synchronous movement on the anthropomorphization of robots. In humans, moving in synchrony has been shown to have a strong impact on the interpersonal relationship and self-other representation. For instance, individuals who synchronized their behavior felt more connected and thought the other was more similar to themselves (Valdesolo et al., 2010). Synchronized behavior also led to increased similarity ratings, compassion, and a higher tendency to display altruistic behavior by helping the person that had been synchronized with (Valdesolo and DeSteno, 2011). We consider the possibility that synchronized movement might not only affect the relationship and perceived similarity between two humans but also between a human and a robot. As we will explain more elaborately below, this might depend on the overlap of the representations of the self and the other. That such representations can be extended to non-biological objects is consistent with findings that non-biological objects can become part of one’s own body representation: Ma and Hommel (2015) have demonstrated ownership illusions for a balloon and a rectangle in the condition in which the object moved in synchrony with the participant’s hand.

We hypothesized that synchronous movement could affect anthropomorphization of a robot in two, opposing, ways. On the one hand, synchrony may increase the perceived similarity between human and robot, because moving in the same way would be an event feature that human and robot would share, and this might increase anthropomorphization—the *feature overlap hypothesis*. On the other hand, however, one may also argue that non-synchronous behavior of a robot increases the perception of its autonomy which, as perceived autonomy (or agency) may contribute to anthropomorphization, may lead to stronger anthropomorphization—the *autonomy hypothesis*.

### The Feature Overlap Hypothesis

The first hypothesis is derived from the Theory of Event Coding (TEC: Hommel et al., 2001), which assumes that the same codes are used to represent perception and action features (Prinz, 1990). Thus, watching someone ride a bike involves the activation of codes that largely overlap with those activated by actually riding a bike oneself. Events are thus represented by networks of feature codes referring to the perceptual and action-related aspects of the event, weighted by the contextual relevance of the involved feature dimensions (Memelink and Hommel, 2013). Two implications of this approach are important for our hypothesis. First, the activation of features follows a pattern-completion logic: if one code of an event representation is activated, activation will spread to the remaining members of the representational code network, so that seeing a bike wheel will not only activate the feature *bike wheel* but will also spread to the codes representing bike, chain, saddle, and pedal, whether these are currently visible or not. Second, TEC does not distinguish between social and non-social events (Hommel et al., 2009), suggesting that it can be applied to humans and non-humans alike.

Combining these two implications allows us to derive a straightforward prediction with respect to the possible impact of synchronous movement. If a human participant and a robot are instructed to move synchronously, as compared to non-synchronously, they share a salient, task-relevant feature. This would render the self-representation of the human and his/her representation of the robot more similar, which should reduce self-robot discriminability. Reducing the discriminability between the representations of two events is likely to allow for feature migration from one representation to the other. For simple objects, this has been first demonstrated by Treisman and Gelade (1980), who found that distracting attention increases the probability of attributing the features of one object to another, simultaneously visible object. Extending the logic of this approach to social situations, Ma et al. (2016) have shown evidence of feature migration from a virtual face that moved in synchrony with the movements of a human participant: in contrast to a condition with non-synchronous movements, the synchrony condition led to more positive mood and better performance in a mood-sensitive creativity task when the avatar started smiling—suggesting that the avatar’s mood migrated to the participant. If we assume that feature migration goes both ways—i.e., features of the other may affect features I associate with myself; features I associate with myself may affect features I associate with the other—it is possible that a synchronously moving robot leads human participants to attribute more human features to the robot, which in turn should lead to stronger anthropomorphization.

Empirical support for this consideration can be found in Morewedge et al. (2007), who demonstrated that when an animal, a robot, or an animated blob move at a speed that is closer to the average human speed of moving, it is anthropomorphized more. Along the same lines, a gender-neutral robot talking in a human-like voice (but not one talking in a robot-like voice) was anthropomorphized more when the gender of the participant matched the gender of the voice (Eyssel et al., 2012). Given that in-group members are seen to overlap with the self more than out-group members (Tropp and Wright, 2001), it is interesting to note another study which showed that a robot was anthropomorphized and liked more when it was presented as in-group, as compared to out-group (Kuchenbrandt et al., 2013). Participants were primed with either a picture of the robot or a picture of a computer, and then had to indicate whether a target word was a primary (e.g., happy) or secondary (e.g., hopeful) emotion, or no emotion at all. When the participants were told that they were in the same group as the robot, being primed with the robot coincided with quicker responses to secondary emotions, than being primed with the computer did. Given that secondary emotions are considered exclusively human (Leyens et al., 2000, 2001), the authors interpreted this as meaning that the in-group robot activated the concept “human.”

### The Autonomy Hypothesis

In their three-factor theory of anthropomorphization, Epley et al. (2007) suggest that unpredictability leads to increased anthropomorphization of a target. Humans generally like to interact effectively with the environment, and this is easier when the environment is predictable. The authors suggest that by attributing human characteristics, such as goals and intentions, to the unpredictably behaving non-human target, people become better able to predict its behavior, and thus resolve the tension between the desire to predict and the actual unpredictability. From that perspective, one might argue that the tendency to anthropomorphize should increase with the degree of unpredictability of another agent. Indeed, Epley et al. (2007) suggest that unpredictability of another agent induces the impression of this agent to be more autonomous—a feature that characterizes humans—which in turn should facilitate the attribution of other human characteristics to the agent. This implies that a robot that moves non-synchronously with a human participant, or that initiates unpredictable movements, should elicit a stronger tendency to anthropomorphize than a robot that moves synchronously with the human.

### The Current Study

To test the feature-overlap hypothesis against the autonomy hypothesis (for the first time, to the best of our knowledge), we exposed human participants to a robot with whom the participants were to interact. This interaction entailed making head movements that were the same as or different from the action partner’s before a computer task that required head movements for responses—thus rendering head movements task-relevant. Three kinds of dependent measures were taken to assess various aspects and implications of anthropomorphization.

First, we used the joint Simon task as a measure of spatial self-other discrimination, and thereby as implicit measure of anthropomorphization. In the regular Simon task (Craft and Simon, 1970; Simon, 1990), one person responds to the identity of one of two different stimuli with a left or right button press on each trial. The stimuli are randomly presented either to the left or the right of a fixation cross, which consistently yields faster and more correct responses if the location of the stimulus and location of the response correspond (the congruent trials)—i.e., the stimulus is presented on the right and the correct response is the right-hand button—than if they do not correspond (the incongruent trials). This difference is called the Simon effect. Interestingly, the effect is also obtained if only one of the two keys is operated by the participant while the other is operated by another agent, whether this is another human being (Sebanz et al., 2003), a wooden hand or a Japanese waving cat (Dolk et al., 2013; Stenzel and Liepelt, 2016). In this version, called the ‘joint Simon task,’ the task is essentially a go/no-go task, requiring a response only when one of the two stimuli appears. The congruency effect in this paradigm is called the ‘joint Simon effect.’ Importantly for our purposes, the joint Simon effect was also obtained in a study where a human participant worked side-by-side with a robot (Stenzel et al., 2012), and the effect was larger when participants were either told that the robot was programmed in a “biologically inspired, autonomous way” than when they were told that it was programmed in a “purely deterministic way.” Another recent study found a joint Simon effect in virtual reality both when the co-actor was a human hand and when it was a robotic hand (Bunlon et al., 2018). Stenzel et al. (2013) similarly found a joint Simon effect for a robotic co-actor, but failed to find a relationship between the size of the effect and explicit self-other inclusion, as measured by asking participants which of six images ranging from widely separated to highly overlapping circles best described the relation between the participant and the robot (the Inclusion of the Other in the Self scale, IOS). The latter is striking from the point of view of the feature overlap hypothesis, though it might be accounted for by the fact that there was no manipulation of self-other similarity. Likewise, Wen and Hsieh (2015) have found a joint Simon effect in participants undergoing fMRI who believed they were performing the task together with a robot, although they found reduced activation in areas associated with thinking about beliefs and intentions of others when compared to neuronal activation of participants who believed they were performing the task with another human. Here too, a manipulation of self-other similarity may have made a difference.

In line with Dolk et al. (2014), we interpret the joint Simon effect as the degree to which the presence of another agent is considered in (i.e., related to) one’s own representation of the task. Since this makes it more difficult to determine whose turn it is on a given trial, the more the other is considered in one’s own task-representation, the greater is the need to distinguish between oneself and the other. An obvious way to distinguish oneself from the other is via location, which makes location task-relevant. This increases attention to location—the feature that produces the Simon effect. Greater self-other similarity, and the subsequent greater reliance on location information that is required to deal with this similarity in order to perform on the task, leads to a more pronounced Simon effect. Hence, a larger joint Simon effect indicates larger self-other similarity, which, according to the feature-overlap hypothesis, is grounds for migration of self-related features to the other, resulting in increased anthropomorphization. However, in a task that requires two agents to take turns, greater self-other similarity might impair response selection even independently from the Simon effect proper. If so, one would not (or not only) expect synchrony between human robots to increase the size of the Simon effect but it may also affect reaction time (RT) in general.

Second, we used the Dictator Game to assess altruism, and thereby as implicit measure of anthropomorphization. Originally a method in experimental economics (Kahneman et al., 1986), the Dictator Game is often used to study fairness, rejection, and altruism, among other things (List, 2007), although it should be noted that other factors such as experimental demand characteristics and social norms play a role as well (Bardsley, 2008). In the Dictator Game, one person is the “dictator” who decides how a given amount of money will be distributed between him- or herself and another player. The other player has no choice but to accept the proposed distribution, hence the term “dictator” to characterize the former player. Since the human and robot were performing the Simon task *together*, and no competitive elements were present nor highlighted, we expected that the participant would consider the robot as a collaborator. Ben-Ner and Kramer (2011) have shown that collaborators are given higher stakes than neutral and competitive opponents, so we expected that more anthropomorphization of the robot would go along with more money given to it. One might object that giving money to a robot could seem counterintuitive (after all, what is it going to use it for?), but previous studies suggest that people are not entirely reluctant to give money to robots (Torta et al., 2013; de Kleijn et al., 2019). Given that synchronization promotes altruism (Valdesolo and DeSteno, 2011), we predicted that synchronized movement with a robot would lead to more money given to it in a one-shot Dictator Game.

Third, three questionnaires were used, two to assess state anthropomorphization (e.g., “Overall, do you believe QBo is capable of having intentions?”; Kozak et al., 2006; Epley et al., 2007; Torta et al., 2013) and one designed to assess trait anthropomorphization (e.g., “To what extent does the average reptile have consciousness?”; Waytz et al., 2010). The state anthropomorphization questionnaires served as explicit measures of anthropomorphization toward the robot, whereas the joint Simon task and Dictator game served as implicit measures of anthropomorphization toward the robot.

In sum, we tested how human participants would be affected by synchronously and non-synchronously moving with a robot in terms of explicit anthropomorphization and implicit measures that would be expected to relate to the degree of anthropomorphization. We distinguished between explicit and implicit measures due to evidence that these may diverge (Kim and Sundar, 2012). Based on the feature-overlap hypothesis, we expected that synchronous movement, compared to non-synchronous movement, would result in a larger joint Simon effect, higher stakes offered in the Dictator Game, and higher state anthropomorphization scores. In contrast, the autonomy hypothesis would predict that synchronous and/or predictable movement (i.e., the robot synchronizing with human-initiated movement) should lead to lower anthropomorphization scores, a smaller joint Simon effect, and lower stakes in the Dictator Game, as compared to non-synchronous and/or unpredictable movement (i.e., the robot moving differently compared to the human-initiated movement, or the robot initiating movements).

## Materials and Methods

### Participants

An *a priori* power analysis using G^∗^Power 3.1.9.2 (Faul et al., 2007) indicated a required sample size of 52 participants, based on an expected effect size of *d* = 0.4, informed by an informal review of the literature. Fifty-four participants were recruited (35 female), most of which (36) were Leiden University students. They were recruited through advertisements, word of mouth, and via e-mail invitations. One participant was excluded from analysis due to evident failure to understand the instructions. The mean age was 23.3 years (total range: 19–30). Inclusion criteria were: healthy adults between 18 and 30 years of age with normal or corrected-to-normal vision. Exclusion criteria were: autism spectrum disorder and the use of psychoactive medication. The study was approved by the Leiden University Psychology Research Ethics Committee. All participants gave written informed consent before participation, following the Declaration of Helsinki, and were given monetary compensation for their time and efforts.

### Manipulation

All participants completed two sessions, during one of which they moved in synchrony with the robot, i.e., mirroring movements, while in the other (order counterbalanced), participants and robot moved non-synchronously, i.e., avoiding mirroring or copying the other’s movements. For half of the participants, the robot was the initiator of the movements in both sessions, with the participant as the follower. The other half of the participants were the initiator themselves in both sessions, with the robot as the follower. This distinction was made because it was thought that there may be differential effects depending on who initiates the movement. No specific direction was predicted. The design resulted in four scenarios: (I) human initiator, synchronous condition; (II) human initiator, non-synchronous condition; (III) robot initiator, synchronous condition; and (IV) robot initiator, non-synchronous condition.

**FIGURE 1:**
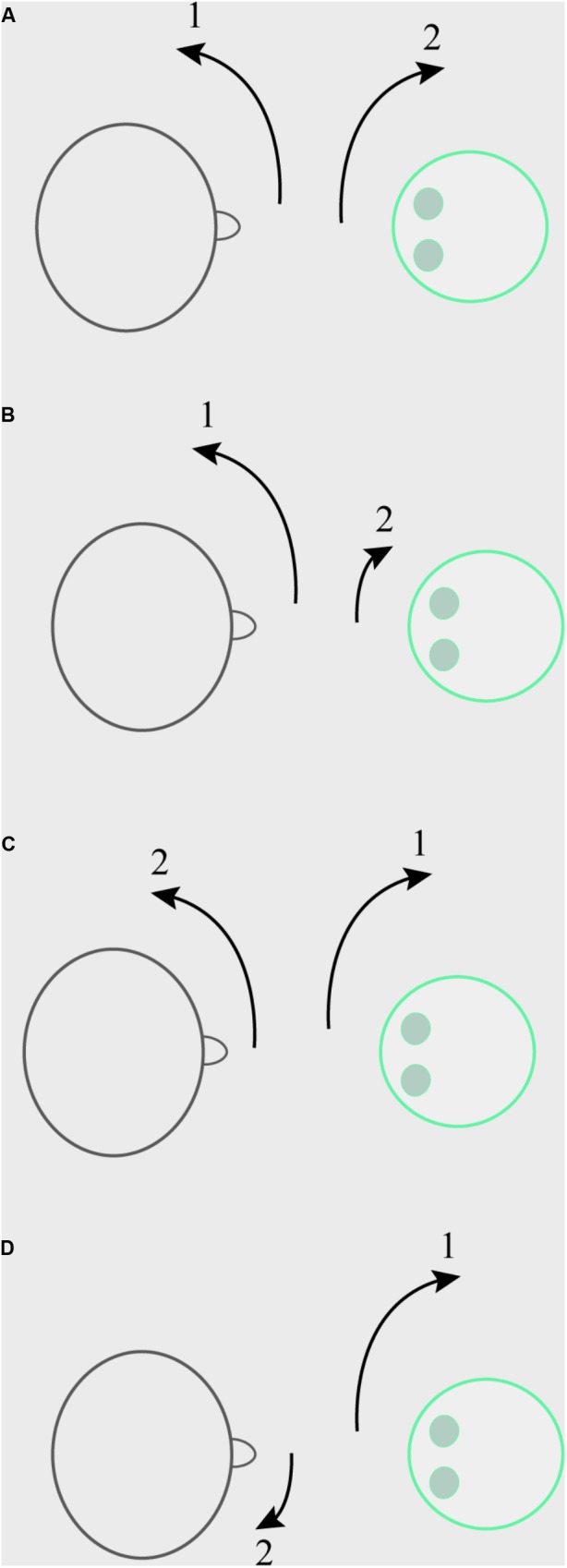
Example of the Synchrony manipulation. **(A)** Human initiator, synchronous condition. The participant (on the left) initiates a large head movement to the left, the robot (on the right) synchronizes with this with a minimal delay by making a large head movement to the right. **(B)** Human initiator, non-synchronous condition. The participant initiates a large head movement to the left, the robot does not mirror this, but makes a small head movement to the right. **(C)** Robot initiator, synchronous condition. The robot initiates a large head movement to the right, with which the participant synchronizes with a minimal delay by making a large head movement to the left. **(D)** Robot initiator, non-synchronous condition. The robot initiates a large head movement to the right, the participant avoids mirroring this by making a small head movement to the right.

In scenario (I), the participant was instructed to start making movements with his or her head, left and right at various speeds and to various degrees, which the robot would then copy. This copying was accomplished by use of a motion tracker sewn onto a cap that the participant wore throughout the session, which communicated with the computer that controlled the robot’s movement. In scenario (II), the participant was instructed to make any of those movements with his/her head, and was told the robot would avoid copying the movements. In scenario (III), the participant was instructed to copy exactly the head movements that the robot made. In scenario (IV), the participant was instructed to avoid moving his/her head in the exact way the robot was at the time the robot was making the movement. The robot’s head movements in these latter three scenarios were randomly generated. It was stressed that in the non-synchronous condition, participants should not make the exact opposite of the robot’s movements, as that is really just like copying. Participants could thus freely move to the opposing or same direction, as long as they moved with a different speed and/or to a different angle compared to the robot at any specific point in time. See Figure 1 for a sketch of the manipulation. Participants either went through scenarios (I) and (II) (those in the human initiator condition), or they went through (III) and (IV) (those in the robot initiator condition), order counterbalanced.

### Measurements

#### Joint Simon Task

**FIGURE 2:**
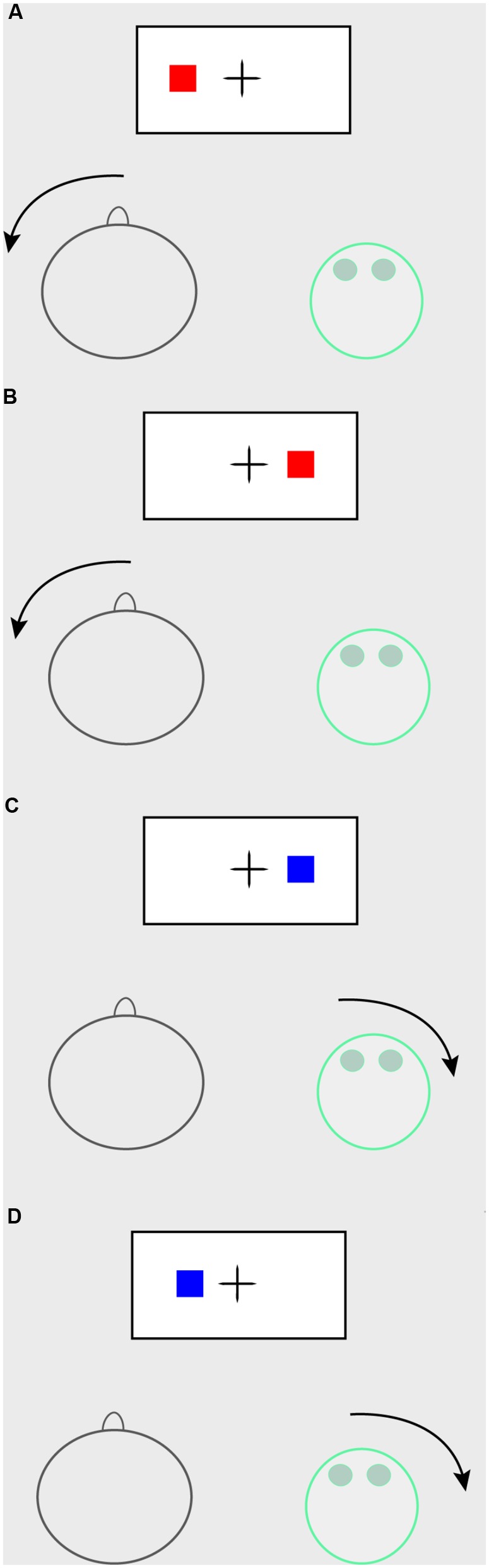
Joint Simon task setup. In this example of the joint Simon task, the participant has to respond to red stimuli, whereas the robot has to respond to blue stimuli. The participant has to respond with a large head movement to the left; the robot has to respond with a large head movement to the right. **(A)** Congruent trial for the participant (stimulus on the participant’s side of the screen); no-go trial for the robot. **(B)** Incongruent trial for the participant (stimulus on the robot’s side of the screen); no-go trial for the robot. **(C)** Congruent trial for the robot (stimulus on robot’s side of the screen); no-go trial for the participant. **(D)** Incongruent trial for the robot (stimulus on the participant’s side of the screen); no-go trial for the participant.

The task was presented on a 21-inch monitor. Each trial started with a fixation cross presented for 500 ms. After this, a blue or a red solid square was presented at either the left or the right of a fixation cross until a response was recorded. Depending on the session’s instructions either the robot or the participant had to respond to the stimulus by turning their head to either the left or the right (see Figure 2). Color and side were counterbalanced between participants, so where one participant may have received the instructions to respond to red squares with a head turn to the right, another may have received instructions to respond to red squares with a head turn to the left, and yet another to respond to blue squares with a head turn to the left. Participants were always informed that the robot would respond to the other color, and with a head motion to the opposite side. Following the response, the next trial was initiated. Participants wore an InterSense InertiaCube4 motion tracker stitched to a cap on their head, which recorded the response onset (i.e., head turn to the left or right) in relation to the stimulus onset in miliseconds, which was used as RT measurement. Participants first completed a practice block of 8 trials, followed by 4 blocks of 64 trials, which made for 256 recorded trials in total.

#### Dictator Game

After the joint Simon Task, participants performed a one-shot Dictator Game with the robot as the opponent. They were presented with a stake, which could be 2, 5, 8, 10, or 20 EUR (randomly drawn each session), and they were asked to decide how much of this stake, if any, they would want to give to the robot. The stakes were varied to control for size, and the outcome measure was the proportion of the stake participants were willing to give to the robot.

#### Questionnaires

At the end of both sessions, participants were asked to fill out three questionnaires: the Individual Differences in Anthropomorphism Questionnaire (IDAQ; Waytz et al., 2010) to assess trait anthropomorphization; the Mind Attribution Scale (MAS, Kozak et al., 2006) to assess state anthropomorphization; and another state anthropomorphization scale taken from Torta et al. (2013), which is based on Epley et al. (2007; henceforth: Torta state questionnaire). The IDAQ trait anthropomorphization questionnaire inquires into general tendency to anthropomorphize, with questions such as “To what extent does a car have free will?”. We expected that people with a high, compared to low, tendency to anthropomorphize would show a larger joint Simon effect and offer more money in the Dicator game, hence we wanted to be able to control for its effects. The state anthropomorphization questionnaires asses anthropomorphization toward something recently interacted with, and were modified to inquire about the robot, TheCorpora’s *QBo* rather than “your opponent” or “this person,” i.e., “Overall, do you believe the opponents you have encountered have free will” became “Overall, do you believe QBo has free will” in the Torta state questionnaire; and “This person has complex feelings” became “QBo has complex feelings” in the MAS. All questionnaires were answered on a 7-point Likert scale. The IDAQ is originally rated on a 10-point Likert scale, but to increase consistency between the questionnaires, and because there is evidence that there is not much difference in answers to Likert scales of seven or more options (Cox III, 1980; Weng, 2004; Dawes, 2008), the response options were reduced to seven. Participants completed all questionnaires in both sessions. After the second session, participants answered five open questions that would give us insight into their experience. All questionnaires can be found in Appendix A.

### The Robot

**FIGURE 3:**
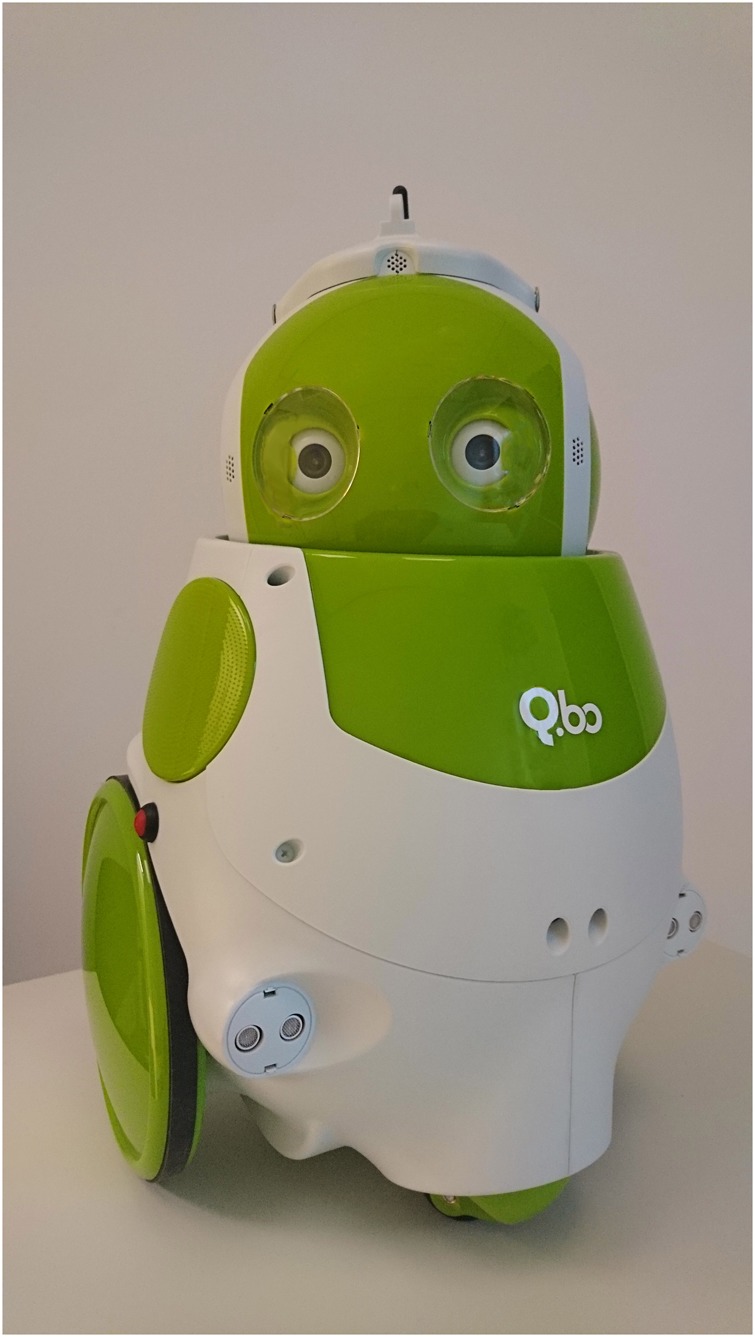
TheCorpora’s QBo. The robot used in our experiment.

TheCorpora’s QBo was used, which is a small, semi-humanoid robot of 45.6 cm high, 31.4 cm wide and 29.25 cm deep, with a curved trunk and round head. It has two large wheels on its sides and one small wheel on the front (reminiscent of a vacuum cleaner), no limbs, but it does have a head that can move in all directions (see Figure 3). The head has two webcams for eyes, a led-light for a nose, and 20 led-lights for a mouth. QBo is mostly white, with elements of green. In scenarios (II), (III), and (IV), the robot received instructions from the computer controlling it for randomly determined head movements. The participant’s motion tracker’s data was disregarded in these scenarios. For scenario (I), the computer controlling the robot received input from the motion tracker, which was translated for the robot to mirror the motion the participant made in realtime. During the joint Simon task, at the start of every trial to which the robot was to respond, a message was sent from the experiment computer (E-Prime in Windows) to the robot computer (Linux) to initiate the appropriate response shortly after stimulus onset. There was no variation in the robot’s response latency, and there were no pre-programmed erroneous responses, although there was a sporadic miscommunication between the computers leading some response omissions on the robot’s end. The number of trials to which the robot failed to respond was not recorded.

### Design and Procedure

The current investigation was a two-session, 2 × 2 mixed design study, with synchrony as within-subjects factor (synchronous vs. non-synchronous) and initiator as between-subjects factor (human initiator vs. robot initiator). Participants completed two sessions of 60 min, 1 week apart. Assignment to conditions and group was performed using http://www.randomization.com/. The study was single-blind: the experiment leader was aware of the condition the participant was in. This was deemed unavoidable due to the novelty of the procedure and the necessity of the experimenter to observe the procedure to ensure it was executed correctly. This could only be accomplished by knowing which movements were required according to the current condition.

At the start of each session, the participant and robot practiced both the synchronous and non-synchronous movements for half a minute, so the participant could experience what the alternative was like. Subsequently they performed the synchrony manipulation that belonged to the current session for 4 min. After every block of 64 trials in the joint Simon task, the manipulation was repeated for 2 min to ensure that the effect did not wear off.

Upon arrival in the first session, participants were informed verbally and by means of an information letter about the study they were about to take part in. After giving written informed consent, they were taken into a room with a one-way mirror, where the experiment took place. The experiment leader was stationed behind the mirror and monitored whether the robot and the program were functioning appropriately, and that the synchronization procedure was executed correctly, not explicitly observing the participant’s behavior for other purposes. Participants were informed of this fact, so as to minimize any effects of observation. The remaining procedure was the same for both sessions, the only difference being the synchronization type. Participants started with a practice session of the synchronization manipulation as explained above, followed by 4 min of the manipulation. After this, they received instructions for the joint Simon task and went through an 8 trial practice block. Thereafter they started on the joint Simon task, with repeats of the manipulation after every block. After the joint Simon task, they completed the one-shot Dictator Game. Finally, they filled out the trait and state anthropomorphization questionnaires. After the second session they filled out some additional questions about their experience of the experiment, which was followed by debriefing and payment.

## Results

### Data Preparation

Before analysis, the data were prepared and filtered in the following way. The Dictator Game offer was coded as a proportion of the total stake, which was used for further analyses.

Principal component analyses with direct oblimin rotation were performed on the three questionnaires to ascertain the structure of the measures. For the Torta state anthropomorphization questionnaire, the Kaiser-Meyer-Olkin (KMO) measure of sampling adequacy was above the recommended 0.6 with a value of 0.864, and the determinant was satisfactory as well (0.065). The communalities were good, ranging from 0.547–0.781. All items were significantly correlated to one another, but none of the correlations were extremely high, indicating there was no reduncancy in the items (range of correlations: 0.479–0.766). The results indicated a one-factor solution, explaining 68.2% of the variance, for the Torta state anthropomorphization questionnaire. Hence, the ratings of each session were added to form one Torta state anthropomorphization score (one for synchronous, one for non-synchronous).

For the MAS state anthropomorphization questionnaire, the KMO was satisfactory as well with a value of 0.839, as was the determinant (0.015). The communalities were good, ranging from 0.508 to 0.758. Most items were significantly correlated (36 out of 45), with a range of 0.177 to 0.655 in correlation coefficients among the significant correlations. The results indicated a two-factor solution, explaining 60.1% of the variance, contrary to the three-factor solution suggested by the authors (Kozak et al., 2006). Factor loadings are presented in the Appendix B. The first factor seemed to be related to ascription of phenomenal consciousness, combining all but one of the items of the emotion and cognition scales that Kozak et al. (2006) identified, whereas the second seemed to reflect ascription of agency, and included one of the cognition scale items (“QBo has a good memory”) in addition to the items that Kozak et al. (2006) found to be in the intentionality scale. Based on these analyses, two MAS scores were calculated for each session: an overall MAS score (for both the synchronous and non-synchronous session), and an agency MAS score (for both the synchronous and non-synchronous session).

Waytz et al. (2010) reported a two-factor solution to best suit the IDAQ items, using both the anthropomorphization and control items in the factor analysis. In our sample, however, a two-factor model explained only 29.6% if the variation. Based on the criterion of Eigenvalue > 1, a 10-factor model emerged, with a low KMO value (0.664), low communalities (ranging from 0.007 to 0.640), and an unsatisfactory determinant (0.0000014), indicating that our sample was not large enough to support this model. It was therefore decided to use only the anthropomorphization items. Based on the criterion of Eigenvalue > 1, a four-factor model emerged explaining 63% of the total variance, one factor representing all items related to technology, one representing all items related to animals, and the items about nature distributed over two factors. A satisfactory KMO value of 0.728 and higher communalities (ranging from 0.295 to 0.829) indicated that this solution was better compared to the model using all items. Waytz et al. (2010) suggested a distinction between anthropomorphization toward animate versus inanimate targets, hence we also ran a two-factor analysis of the data. The animate versus inanimate distinction was, however, not reflected in the two-factor model, nor was any other pattern evident. This model explained only 46.3% of the variance. The KMO was satisfactory (0.728), but the communalities were lower (ranging from 0.179 to 0.744). All things considered, a three-factor model seemed to most sensibly capture the data, one factor representing items related to technology, one representing items related to animals, and one representing items related to nature, in total explaining 56.1% of the variance. KMO was satisfactory (0.728), as was the determinant (0.002), and the communalities were better than for the two-factor model (ranging from 0.293 to 0.744). Having established the structure of the questionnaire, and having assured that all items contributed to the scale, a total IDAQ score was computed for each session.

The session 1 (*M* = 41.7, *SD* = 9.6) and session 2 (*M* = 40.8, *SD* = 10.4) IDAQ trait scores were combined and averaged, assuming that the average of two moments in time of filling in a questionnaire gives a better indication of general anthropomorphization tendencies than does a single one, and the resulting score was used in further analyses. Indeed, the correlation between the two showed a good test-retest reliability [*r*(53) = 0.841, *p* < 0.001]. Please refer to Table 1 for descriptive statistics for all measures.

**Table 1:** Descriptive statistics.

Initiator	Outcome measure	Session	Mean	Std. Deviation	Minimum	Maximum
Human	IDAQ mean trait		46.52	10.82	26.00	67.50
	Torta state	Nonsynchronous	8.81	4.40	5.00	22.00
		Synchronous	8.07	3.59	5.00	17.00
	MAS state	Nonsynchronous	28.63	9.32	10.00	51.00
		Synchronous	26.30	9.93	10.00	47.00
	Response time congruent	Nonsynchronous	423.74	68.07	281.22	542.12
		Synchronous	433.13	63.41	342.61	596.55
	Response time incongruent	Nonsynchronous	439.83	75.65	295.00	578.24
		Synchronous	445.79	70.06	339.08	604.59
	Joint Simon effect	Nonsynchronous	16.41	18.65	–15.91	49.85
		Synchronous	12.67	22.20	–26.64	55.26
	Dictator Game offer	Nonsynchronous	0.29	0.26	0.00	0.80
		Synchronous	0.33	0.28	0.00	1.00
Robot	IDAQ mean trait		47.44	7.89	30.00	65.00
	Torta state	Nonsynchronous	8.96	5.24	5.00	24.00
		Synchronous	8.69	4.32	5.00	20.00
	MAS state	Nonsynchronous	28.19	9.30	10.00	50.00
		Synchronous	28.04	8.18	15.00	46.00
	Response time congruent	Nonsynchronous	442.06	55.55	347.75	567.58
		Synchronous	428.21	60.55	361.02	581.51
	Response time incongruent	Nonsynchronous	459.45	64.67	360.19	614.49
		Synchronous	441.77	57.00	377.78	591.65
	Joint Simon effect	Nonsynchronous	17.39	29.54	–34.10	103.04
		Synchronous	13.56	21.75	–21.07	51.18
	Dictator Game offer	Nonsynchronous	0.32	0.31	0.00	1.00
		Synchronous	0.24	0.23	0.00	0.50

The data of the Torta questionnaire, the joint Simon task, and the Dictator Game did not meet the assumption of normally distributed residuals. A log transformation did not sufficiently eliminate this problem. However, given that there is evidence that ANOVAs are robust against violations of this assumption (Blanca et al., 2017a,b), the planned mixed ANOVAs were performed. To determine the mean RTs, we used a recursive outlier detection method with a moving criterion (Selst and Jolicoeur, 1994), which has been shown to be relatively insensitive to sample size and skew compared to non-recursive methods (e.g., 2.5 SD from the mean) and recursive methods without moving criterion. This is appropriate due to the natural skewness of RT data.

To determine whether IDAQ trait anthropomorphization interacted with the independent variables and thus whether it could be used as a covariate for the mixed ANOVAs on RT, Dictator Game offer, and the state anthropomorphization questionnaires, we performed linear regression analyses. We ran separate analyses per outcome variable and per timepoint (synchrony condition), and used IDAQ and the interaction between initiator and IDAQ as predictors. For each of the analyses, the interaction was significant [RT_synchronous_: *F*(3,6036) = 134.345, *p* < 0.001, *R*^2^ = 0.063; RT_non-synchronous_: *F*(3,6042) = 95.722, *p* < 0.001, *R*^2^ = 0.045; DG_synchronous_: *F*(3,49) = 144.786, *p* < 0.001, *R*^2^ = 0.899; DG_non-synchronous_: *F*(3,49) = 172.051, *p* < 0.001, *R*^2^ = 0.913; MAS_synchronous_: *F*(3,49) = 155.929, *p* < 0.001, *R*^2^ = 0.905; MAS_non-synchronous_: *F*(3,49) = 159.343, *p* < 0.001, *R*^2^ = .907; Torta_synchronous_: *F*(3, 49) = 229.920, *p* < 0.001, *R*^2^ = 0.934; Torta_non-synchronous_: *F*(3,49) = 191.557, *p* < 0.001, *R*^2^ = 0.921], meaning the intended covariate was not independent, thus it could not be added without violating the assumption. We therefore ran the mixed ANOVAs without IDAQ trait anthropomorphization, and added Spearman correlation analyses to inquire into the relationship of IDAQ trait anthropomorphization and the dependent variable. The correlations reported below are all Spearman rho’s (*ρ*), since they all involve questionnaire data. Results of all mixed ANOVAs described below are displayed in Table 2. All mixed ANOVAs were backed up by Bayesian mixed ANOVAs, the results of which are to be found in Table 3.

**Table 2:** Results for all mixed ANOVAs.

Dependent variable	Predictor	*F*	*p*	$\eta_{p}^{2}$
Joint Simon effect	Synchrony	0.791	0.378	0.015
	Congruency	30.306	0.000	0.373
	Synchrony ^∗^ initiator	7.148	0.010	0.123
	Congruency ^∗^ initiator	0.060	0.807	0.001
	Synchrony ^∗^ congruency	0.953	0.333	0.018
	Synchrony ^∗^ congruency ^∗^ initiator	0.015	0.903	0.000
Dictator Game offer	Synchrony	0.627	0.432	0.012
	Synchrony ^∗^ initiator	3.496	0.067	0.064
Torta	Synchrony	2.037	0.160	0.038
	Synchrony ^∗^ initiator	0.444	0.508	0.009
MAS	Synchrony	2.335	0.133	0.044
	Synchrony ^∗^ initiator	1.793	0.186	0.034

**Table 3:** Bayes Factors for inclusion of specified terms compared to models without those terms.

Dependent variable	Predictor	BF inclusion
Joint Simon effect	Initiator	0.577
	Synchrony	0.278
	Congruency	2843.892
	Synchrony ^∗^ initiator	111.516
	Congruency ^∗^ initiator	0.191
	Synchrony ^∗^ congruency	0.247
	Synchrony ^∗^ congruency ^∗^ initiator	0.285
Dictator Game offer	Initiator	0.400
	Synchrony	0.258
	Synchrony ^∗^ initiator	1.187
Torta	Initiator	0.500
	Synchrony	0.508
	Synchrony ^∗^ initiator	0.338
MAS	Initiator	0.469
	Synchrony	0.594
	Synchrony ^∗^ initiator	0.550

### Joint Simon Effect

**FIGURE 4:**
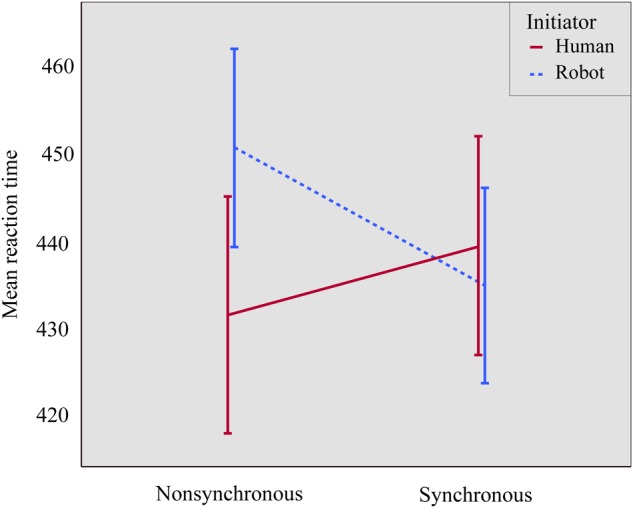
The effect of the interaction of synchrony and initiator on RT. Error bars represent standard errors. Participants in the human initiator group were faster in the non-synchronous compared to the synchronous condition (*M*_synchronous_ = 439, *SD*_synchronous_ = 11.9; *M*_non-synchronous_ = 432, *SD*_non-synchronous_ = 12.6), whereas participants in the robot initiator group were faster in the synchronous compared to the non-synchronous condition (*M*_synchronous_ = 435, *SD*_synchronous_ = 12.2; *M*_non-synchronous_ = 451, *SD*_non-synchronous_ = 12.8).

We ran a mixed ANOVA on mean RT, with two within-subjects factors (synchrony: synchronous vs. non-synchronous; and congruency: congruent vs. incongruent) and one between-subjects factor (initiator: robot vs. human). There was a significant main effect of congruency [*F*(1,51) = 30.306, *p <* 0.001, $\eta_{p}^{2}$ = 0.373], where responses on congruent trials (*M* = 431, *SD* = 8.2) were faster than on incongruent trials (*M* = 446, *SD* = 9.0), thus replicating the joint Simon effect with a robotic partner. Additionally, there was a significant synchrony ^∗^ initiator interaction [*F*(1,50) = 7.148, *p* = 0.01, $\eta_{p}^{2}$ = 0.123], where those in the human initiator group had shorter RTs in the non-synchronous (*M* = 432, *SD* = 12.6) than in the synchronous condition (*M* = 439, *SD* = 11.9), whereas those in the robot initiator group had shorter RTs in the synchronous (*M* = 435, *SD* = 12.2) than in the non-synchronous condition (*M* = 451, *SD* = 12.8), see Figure 4. Follow-up paired *t*-tests showed that this effect was driven by a significant synchrony effect in the robot initiator group [*t*(25) = 2.644, *p* = 0.014]; the difference did not reach significance in the human initiator group [*t*(26) = 1.212, *p* = 0.236]. Notably, a number of participants in the robot initiator group reported difficulty during the non-synchronous session’s manipulation. They found it taxing to simultaneously monitor the robot’s movements, plan their own movements, and make sure they were not the same. However, as this was spontaneous self-report, and not systematically assessed, we cannot take this into account in analyses.

To provide stronger evidence for the current results, a Bayesian mixed ANOVA with the same factors was conducted. To determine which effects are likely predictors of RT, we looked at the Bayes Factors for addition of each of the terms to a model without that specific term (Jarosz and Wiley, 2014; Wagenmakers et al., 2018), i.e., Inclusion Bayes Factor based on matched models. The results concur with the regular mixed ANOVA, and provided very strong evidence that congruency (BF_inclusion_ = 2843.892) and the synchrony ^∗^ initiator interaction (BF_inclusion_ = 111.516) were predictors in explaining the RT data.

The correlation between the congruency effect (synchronous and non-synchronous averaged) and IDAQ trait anthropomorphization was not significant [*ρ*(53) = 0.187, *p* = 0.181], indicating that there was no relationship between baseline tendency to anthropomorphize and the congruency effect. Similarly, and following up on the synchrony ^∗^ initiator interaction, there was no significant correlation between the synchrony effect (average synchronous RT – average non-synchronous RT) and the IDAQ trait anthropomorphization for either the human or robot initiator group [human initiator: *ρ*(27) = 0.237, *p* = 0.234; robot initiator: *ρ*(26) = -0.025, *p* = 0.904].

We also looked into the correlations between the intention subscale of the MAS state anthropomorphization questionnaire and the joint Simon effect for the synchronous and non-synchronous sessions, since this subscale seems to give an indication of ascription of agency to the robot (“QBo is capable of doing things on purpose”; “QBo is capable of planned action”; “QBo has goals”; “QBo has a good memory”). There was no significant correlation [synchronous: *ρ*(53) = 0.121, *p* = 0.389; non-synchronous: *ρ*(53) = -0.099, *p* = 0.480], indicating that there was no relationship between ascription of agency and congruency effect. The same held for the correlations between the subscale of the MAS and the overall RTs, indicating that the interaction reported above was not driven by (explicit) ascription of agency.

Finally, we looked into order effects, since the nature of the initial interaction with the robot may affect the perception of the robot in the later session: it may be sticky, so to speak. To this end, we performed the mixed ANOVA on mean RT as above, with synchrony and congruency as within-subjects factors, and in addition to initiator, also order (synchronous-first vs. non-synchronous-first) as between-subjects factor. Order was involved in a significant interaction with congruency and synchrony [*F*(1,49) = 6.240, *p* = 0.016, $\eta_{p}^{2}$ = 0.113], where both order groups showed a numerical decrease in joint Simon effect in the second session, which was rather pronounced in the group that had the non-synchronous session first (session 1: *M* = 20.33, *SD* = 26.9; session 2: *M* = 8.50, *SD* = 19.3), and rather negligible in the group that had the synchronous session first (session 1: *M* = 17.54, *SD* = 23.4; session 2: *M* = 12.80, *SD* = 21.5). However, a follow-up paired *t*-test comparing the session 1 and session 2 joint Simon effects for both of the order groups showed that the difference was non-significant in both cases [synchronous first: *t*(26) = 1.463, *p* = 0.156; non-synchronous first: *t*(25) = 2.048, *p* = 0.051], so that we are reluctant to interpret the interaction.

### Dictator Game

A mixed ANOVA on Dictator Game offer was performed, with one within-subjects factor (synchrony: synchronous vs. non-synchronous) and one between-subjects factor (initiator: robot vs. human). There were no significant effects, all *p*s > 0.067. The correlation between IDAQ and the difference between the DG offers (synchronous – non-synchronous) was not significant [*ρ*(53) = -0.103, *p* = 0.462], indicating that there was no relationship between tendency to anthropomorphize and altruism toward the robot. To confirm that the offer did not differ as a result of our manipulation, we conducted a Bayesian mixed ANOVA. The results indicate weak support for the synchrony ^∗^ initiator interaction (BF_inclusion_ = 1.187).

As with the joint Simon effect, we looked at the correlations between the intention subscale of the MAS state anthropomorphization questionnaire and the Dictator Game offer for the synchronous and non-synchronous sessions. There was no significant correlation [synchronous: *ρ*(53) = 0.265, *p* = 0.055; non-synchronous: *ρ*(53) = 0.223, *p* = 0.109], indicating that there was no relationship between ascription of agency and proportion offered in the Dictator Game.

The Dictator Game offer thus did not vary as a function of our manipulation. It did, however, correlate positively with anthropomorphization of the robot: the offer on the synchronous session correlated positively with both state anthropomorphization questionnaires [MAS: *ρ*(53) = 0.361, *p* = 0.008; Torta: *ρ*(53) = 0.423, *p* = 0.002], and the offer on the non-synchronous session correlated positively with the Torta state anthropomorphization questionnaire [*ρ*(53) = 0.336, *p* = 0.014]. There were no correlations with the MAS intention subscale, suggesting that this relationship did not depend on perceived autonomy.

Finally, we looked into order effects by running the aforementioned mixed ANOVA with synchrony as within-subjects factor, and initiator and order as between-subjects factors. Order was not a significant contributor, indicating that the Dictator Game offer was not affected by the order of the manipulation.

### Trait Anthropomorphization

The IDAQ trait anthropomorphization questionnaire showed good internal consistency (α_synchronous_ = 0.724; α_non-synchronous_ = 0.760) in addition to the aforementioned good test-retest reliability.

An independent samples *t*-test was run to compare the two initiator groups on IDAQ trait anthropomorphization, to make sure there were no baseline differences between the groups. The result was non-significant [*M*_human_ = 41.0, *SD*_human_ = 11.1; *M*_robot_ = 41.5, *SD*_robot_ = 8.0; *t*(51) = 0.180, *p* = 0.858], meaning there were indeed no baseline differences in terms of tendency to anthropomorphize between the two initiator groups.

### State Anthropomorphization

The Torta state anthropomorphization questionnaire showed high internal consistency (α_synchronous_ = 0.787; α_non-synchronous_ = 0.892). A mixed ANOVA on the Torta state anthropomorphization questionnaire was performed, with one within-subjects factor (synchrony: synchronous vs. non-synchronous) and one between-subjects factor (initiator: robot vs. human). There were no significant effects, meaning that state anthropomorphization as measured by the Torta questionnaire did not differ as a result of our manipulation.

To confirm that Torta state anthropomorphization did not vary as a function of our manipulation, we ran a Bayesian mixed ANOVA with the same factors as above. The inclusion Bayes Factors were very low (all below 0.508), indicating that the null model was the best explanation of the data, and confirming that this measure did not vary as a function of our manipulation.

Here too we looked into order effects by running the above mixed ANOVA with synchrony as within-subjects factor, and initiator and order as between-subjects factors. The absence of significant effects of order indicated that the order of the manipulation did not affect anthropomorphization of the robot as measured by the Torta state anthropomorphization questionnaire.

The MAS state anthropomorphization questionnaire showed high internal consistency (α_synchronous_ = 0.801; α_non-synchronous_ = 0.824). A mixed ANOVA on the MAS state anthropomorphization questionnaire was performed, with one within-subjects factor (synchrony: synchronous vs. non-synchronous) and one between-subjects factor (initiator: robot vs. human). This too yielded no significant effects, indicating that state anthropomorphization as measured by the MAS did not differ as a result of our manipulation.

To confirm that the MAS state anthropomorphization questionnaire did not vary as a function of our manipulation, we ran a Bayesian mixed ANOVA with the same factors as above. The inclusion Bayes Factors were very low (all below 0.594) in this case as well, indicating that the null model was the best explanation of the data. Neither of the state anthropomorphization questionnaires thus showed an effect of the manipulation.

Order effects were examined by running a mixed ANOVA on the MAS state anthropomorphization questionnaire scores with synchrony as within-subjects factor, and initiator and order as between-subjects factors. There was a significant synchrony ^∗^ order interaction [*F*(1,49) = 16.967, *p* < 0.001, $\eta_{p}^{2}$ = 0.257]. For both order groups, the robot was numerically anthropomorphized less in the second session. This seemed to be more pronounced in the non-synchronous-first group (session 1: *M* = 28.73, *SD* = 6.2; session 2: *M* = 24.50, *SD* = 7.0) compared to the synchronous-first group (session 1: *M* = 29.70, *SD*: 10.2; session 2: 28.11, *SD* = 11.6). A follow-up *t*-test indicated that this was significant in the non-synchronous-first group [*t*(25) = 3.784, *p* = 0.001], while it failed to reach significance in the synchronous-first group [*t*(26) = 1.736, *p* = 0.094]. Hence, while the previous questionnaire was not affected by the order of the manipulation, the MAS state anthropomorphization questionnaire was. This may be explained by a difference in the two questionnaires; while they have largely overlapping items, the MAS state anthropomorphization questionnaire has some additional items that are not captured by the Torta state anthropomorphization questionnaire (“QBo is capable of planned actions”; “QBo has a good memory,” “QBo can engage in a great deal of thought,” and “QBo has goals”), which might be interpreted as related to rational cognitive function.

A correlation analysis was performed with IDAQ trait anthropomorphization and each of the state anthropomorphization scores (synchronous and non-synchronous scores for each questionnaire separately). The IDAQ was positively correlated to each [Torta_synchronous_: *ρ*(53) = 0.595, *p* < 0.001; Torta_non-synchronous_: *ρ*(53) = 0.584, *p* < 0.001; MAS_synchronous_: *ρ*(53) = 0.373, *p* = 0.006; MAS_non-synchronous_: *ρ*(53) = 0.392, *p* = 0.004], meaning that the higher the tendency to anthropomorphize, the higher the actual anthropomorphization of the robot in the experiment.

## Discussion

This study investigated whether anthropomorphization of a robot could be influenced by moving with it either synchronously or non-synchronously, and whether this would be affected by who initiated the movements. We pitted two hypotheses against each other: the feature-overlap hypothesis and the autonomy hypothesis. The former predicted that the robot would be anthropomorphized more following synchronous movement while the latter predicted the robot would be anthropomorphized more following unpredictable movement, i.e., non-synchronous when the human initiated the movements, or either synchrony condition when the robot initiated the movements.

In the joint Simon task, we replicated the joint Simon effect with a robotic co-actor, concurrent with previous studies (Stenzel et al., 2012; Stenzel et al., 2013; Wen and Hsieh, 2015; Bunlon et al., 2018). Contrary to expectations, the size of the joint Simon effect was not affected by our manipulation. The manipulation did, however, affect RTs overall: for the group in which the human initiated the movements, the RTs were larger when the robot synchronized with the human than when the robot did not synchronize with the human. Conversely, for the group in which the robot initiated the movements, the RTs were larger when the human was instructed not to synchronize with the robot compared to when the human was told to synchronize with it. This pattern of results fits neither of the advanced hypotheses. The autonomy hypothesis would have predicted the opposite pattern in the group in which the human initiated the movements, and additionally there should not have been a difference in the group in which the robot initiated the movements—which there is. Additionally, there was no relationship between the questionnaire items assessing ascription of agency and the joint Simon effect, nor with overall RT, which leaves the autonomy hypothesis with even less support.

The feature overlap hypothesis would have predicted the increase in RT in the synchronous condition when the human initiated the movements, but would have also predicted to find this in the group in which the robot initiated the movements. It thus seems that neither hypothesis is sufficient to explain the results. Perhaps they can be explained by a difference in difficulty between the manipulations: In the human initiator condition, participants could safely ignore the behavior of the robot, which also did not overlap with their own action or action planning. In the robot initiator condition, however, they had to take the behavior of the robot into account, and it makes sense to assume that this required less cognitive effort in the synchrony as compared to the non-synchronous condition, where the behavior had to be mentally “inverted” to specify one’s own action plan. This may have made the non-synchronous condition cognitively incompatible, which is known to impair action planning and response selection (Proctor and Vu, 2006). The potential asymmetry in the manipulation in terms of difficulty is therefore an unforeseen shortcoming of the current design.

The Dictator Game offer seemed entirely unaffected by our manipulation. Like the joint Simon task, there was no relationship with ascription of agency either. There was, however, a correlation between anthropomorphization of the robot and the size of the offer: the more the participant anthropomorphized the robot, the larger the proportion of the stake the participant offered. While this is consistent with previous findings suggesting a connection between trust and anthropomorphization (Hancock et al., 2011), it does not suggest a moderating role of synchrony. It may shed some light on inconsistent findings reported by Müller et al. (2014). After watching either a fragment of Pinocchio or a Dutch romantic comedy in one study, and after watching a fragment of Pinocchio or a documentary in which a wooden puppet is made in another study, participants were asked to choose a seat in a row of chairs with a wooden doll on the one end and a backpack (implying a human) on the other end, and were then asked to distribute seven lottery tickets worth €5 each between the human and a wooden puppet. In the second study, participants then also filled in a few questions about their perception of Pinocchio. In both studies, they found that participants sat closer to the wooden doll following the Pinocchio fragment compared to the other fragment. Additionally, they found an effect of movie fragment upon distribution of money in the former (with seating distance as covariate), but not in the latter study. Finally, they report negative correlations between seating distance and ascription of intentionality and will to the wooden doll, indicating that the more the participant perceived the doll as having an own will and intentionality, the closer they decided to sit to it.

To link these findings to the current study, two things are to be noted. (i) The studies differ in that in the former, only those in the Pinocchio fragment condition are exposed to a wooden doll prior to selecting the chairs, whereas in the latter study, both groups of participants are exposed to a wooden doll. (ii) The negative correlations reported pertain to the whole sample, thus not only to those in the condition in which the wooden doll might be expected to be perceived more human-like (i.e., the Pinocchio fragment). Linking our findings to (i), in our study, all participants were exposed to the robot in both sessions, rendering our design analogous to the second experiment. One explanation of our nullfindings based on synchronization condition thus is that exposure to the robot is all that determines altruism toward it. Since exposure is equal, no difference is to be expected. Linking our findings to (ii), the Müller et al. (2014) study leaves open the possibility that anthropomorphization of the wooden doll affects the amount of money allocated to it: they report that higher ascription of agency relates to closer seating next to the doll; and since closer seating next to the doll is taken as covariate in analyzing the allocation of money, possible variation due to anthropomorphization of the doll is taken out. Hence, their findings may be taken together with ours to suggest that mere exposure as well as baseline tendency to anthropomorphize affect altruism toward inanimate objects, so that differences in altruism can be found when comparing differential exposure to and/or differences in levels of anthropomorphization of the inanimate object.

Our manipulation had no effect on explicit anthropomorphization of the robot, as indicated by a lack of difference on the state anthropomorphization measures (Kozak et al., 2006; Torta et al., 2013). We did, however, find that a higher tendency to anthropomorphize (as measured by the IDAQ, Waytz et al., 2010) translated into more actual anthropomorphization of the robot, lending credibility to both measures. Additionally, we have found all questionnaires to have good internal consistency, and have found that the trait anthropomorphization questionnaire showed good test-retest reliability.

Interestingly, there was some indication that the order in which the synchronization manipulations were experienced affected anthropomorphization of the robot. Although this was not the case for all measures, the joint Simon effect and the MAS state anthropomorphization questionnaires showed an order effect that followed a similar pattern: anthropomorphization was reduced in the second as compared to the first session, and this was particularly so for the group that had the non-synchronous session first. We may draw two tentative conclusions from this: that more exposure to the robot does not lead to more anthropomorphization and that having had a non-synchronous interaction before a synchronous interaction leads to a stronger reduction of anthropomorphization in the latter.

Where do the results leave the two possible hypotheses? Unfortunately, it seems that either our manipulation was not ideal for testing the hypotheses, or that neither of the mechanisms has an effect: most measures showed similar results across conditions. There was an effect of our manipulation on RTs in the joint Simon task, but this may have been due to a difference in difficulty of the manipulation. The agency-related items of one of the questionnaires did not relate to this effect, leaving that hypothesis with less support—at least at the level of self-report. However, since we have not used a similar measure of self-reported self-other overlap—a shortcoming of the current design—we cannot make any similar claims about the feature-overlap hypothesis. Other possible reasons for the lack of an effect include sample size, the distinct non-human appearance of the robot, and more interestingly: the motion patterns of the robot. The movement of the robot, though superficially mimicking human motion, has a monotonic speed, whereas human (and other biological) motion does not. On the one hand, previous findings do not suggest that monotonic speed as such stands in the way of social interactions with robots: for instance, van den Brule et al. (2014) found no impact of motion style on the trustworthiness of robotic agents. On the other hand, however, our synchrony manipulation might have increased the salience of the non-biological nature of the robotic movements, which in turn might have emphasized the perceived dissimilarity between the human and the robot. Future studies might overcome this possible obstacle by using humanoid robots programmed to move in a more biologically plausible way. For the time being, however, our findings do not point to a strong role of behavioral synchrony in human-robot interaction.

## Data Availability Statement

Datasets are available on request.

## Author Contributions

All authors developed the study concept, contributed to the study design, and provided critical revisions to and approved the final version of the manuscript. SH programmed the tasks, collected and analyzed the data, and drafted the manuscript. RdK programmed the robot and motion tracker.

## Conflict of Interest Statement

The authors declare that the research was conducted in the absence of any commercial or financial relationships that could be construed as a potential conflict of interest.
