# Supplementary material for: The Impact of Human–Robot Synchronization on Anthropomorphization
Source: Front Psychol. 2019 Jan 8;9:2607. doi: 10.3389/fpsyg.2018.02607 (PMC6331525; doi:10.3389/fpsyg.2018.02607)
Supplement: Supplementary file 1 [file Table_1.DOCX]

**Appendix A: Anthropomorphization questionnaires**

1. IDAQ, Waytz, Cacioppo & Epley, 2010 (trait)

To what extent is the desert lethargic (sleepy, lazy)?

To what extent is the average computer active?

To what extent does technology (devices and machines for manufacturing, entertainment, and productive processes) have intentions?

To what extent does the average fish have free will?

To what extent is the average cloud good-looking?

To what extent are pets useful?

To what extent does the average mountain have free will?

To what extent is the average amphibian lethargic (sleepy, lazy)?

To what extent does a television experience emotions?

To what extent is the average robot good-looking?

To what extent does the average robot have consciousness?

To what extent do cows have intentions?

To what extent does a car have free will?

To what extent does the ocean have consciousness?

To what extent is the average camera lethargic (sleepy, lazy)?

To what extent is a river useful?

To what extent does the average computer have a mind of its own (the ability to make its own decisions)?

To what extent is a tree active?

To what extent is the average kitchen appliance useful?

To what extent does a cheetah experience emotions?

To what extent does the environment experience emotions?

To what extent does the average insect have a mind of its own (the ability to make its own decisions)?

To what extent does a tree have a mind of its own (the ability to make its own decisions)?

To what extent is technology (devices and machines for manufacturing, entertainment and productive processes) durable (duurzaam)

To what extent is the average cat active?

To what extent does the wind have intentions?

To what extent is the forest durable (duurzaam)?

To what extent is a tortoise durable (duurzaam)?

To what extent does the average reptile have consciousness?

To what extent is the average dog good-looking?

*Response options*: Not at all – A tiny bit – A bit – Some – Quite a bit – A lot – Very much.

1. Anthropomorphization scale based on Epley 2007, taken from Torta et al., 2013 (state). Modified to fit our study.

Overall, do you believe QBo is capable of feeling emotions?

Overall, do you believe QBo is capable of having intentions?

Overall, do you believe QBo has consciousness?

Overall, do you believe QBo has a mind of his own?

Overall, do you believe QBo has free will?

*Response options*: Not at all – A tiny bit – A bit – Some – Quite a bit – A lot – Very much.

1. Mind Attribution Scale, Kozak, Marsh & Wegner, 2006. Modified to fit our study.

QBo has complex feelings

QBo can experience pain

QBo is capable of emotion

QBo can experience pleasure

QBo is capable of doing things on purpose

QBo is capable of planned actions

QBo has goals

QBo is highly conscious

QBo has a good memory

QBo can engage in a great deal of thought

*Response options*: Strongly disagree – Mostly disagree – Somewhat disagree – Neutral – Somewhat agree – Mostly agree – Strongly agree.

1. Debriefing questions (open questions)

What do you think we are trying to study? (Hypothesis)

Have you participated in any other robot or virtual reality experiments? If so, will you please briefly tell us when, how often, and what kind?

What did you think of QBo, our robot, when you first saw him?

What do you think of QBo now?

Do you have any remarks, or things you'd like to tell/ask us about our study?

**Appendix B: Factor analysis results**

| Table B1. Factor loadings for the Torta state anthropomorphization questionnaire | |
| --- | --- |
|  | Component |
|  | 1 |
| Overall, do you believe QBo has free will? | **.884** |
| Overall, do you believe QBo has a mind of his own? | **.845** |
| Overall, do you believe QBo is capable of feeling emotions? | **.840** |
| Overall, do you believe QBo has consciousness? | **.813** |
| Overall, do you believe QBo is capable of having intentions? | **.739** |

| Table B2. Factor loadings for the Mind Attribution Scale | |  | |  |
| --- | --- | --- | --- | --- |
|  | | Component | | |
|  | 1  *Consciousness* | | 2  *Agency* | |
| QBo is capable of emotion | **.878** | | -.022 | |
| QBo has complex feelings | **.870** | | -.121 | |
| QBo can experience pain | **.815** | | -.047 | |
| QBo can engage in a great deal of thought | **.747** | | .016 | |
| QBo is highly conscious | **.729** | | .101 | |
| QBo can experience pleasure | **.713** | | .141 | |
| QBo is capable of doing things on purpose | -.023 | | **.765** | |
| QBo has goals | .144 | | **.744** | |
| QBo has a good memory | .012 | | **.709** | |
| QBo is capable of planned actions | -.060 | | **.676** | |

| Table B3. Factor loadings for the IDAQ | | | |
| --- | --- | --- | --- |
|  | Component | | |
|  | 1  *Nature* | 2  *Animals* | 3  *Technology* |
| To what extent does the average mountain have free will? | **.883** |  |  |
| To what extent does the ocean have consciousness? | **.843** |  |  |
| To what extent does the environment experience emotions? | **.522** | -.118 | .195 |
| To what extent does the wind have intentions? | **.520** |  | .422 |
| To what extent does a tree have a mind of its own? | **.444** | .226 |  |
| To what extent does the average fish have free will? |  | **.838** |  |
| To what extent does the average insect have a mind of its own? | .209 | **.808** |  |
| To what extent do cows have intentions? |  | **.802** | -.130 |
| To what extent does the average reptile have consciousness? | .136 | **.780** |  |
| To what extent does a cheetah experience emotions? |  | **.703** | .120 |
| To what extent does the average robot have consciousness? |  |  | **.852** |
| To what extent does technology—devices and machines—have intentions? |  |  | **.776** |
| To what extent does the average computer have a mind of its own? |  | .128 | **.738** |
| To what extent does a car have free will? | .216 |  | **.533** |
| To what extent does a television set experience emotions? | .113 | -.166 | **.467** |
